# Paraneoplastic Myeloperoxidase (MPO)-Antineutrophil Cytoplasmic Antibody (ANCA)-Associated Vasculitis Revealing Colon Adenocarcinoma in a Patient With Controlled HIV Infection: A Case Report

**DOI:** 10.7759/cureus.109274

**Published:** 2026-05-20

**Authors:** Leidy P Cespedes Useche, Sergio Bolivar, Oscar I Reyes, Daniel Rodriguez-Peralta, Diana Sanchez

**Affiliations:** 1 Internal Medicine, Central Military Hospital, New Granada Military University, Bogotá, COL; 2 Hematology and Medical Oncology, Central Military Hospital, Bogotá, COL; 3 Nephrology, Central Military Hospital, Bogotá, COL

**Keywords:** anca-associated vasculitis, colon adenocarcinoma, hiv infection, mpo-anca, paraneoplastic syndrome

## Abstract

The relationship between antineutrophil cytoplasmic antibody (ANCA)-associated vasculitis (AAV) and solid malignancies is uncommon and poses significant diagnostic challenges, particularly in individuals with underlying immune dysregulation, such as human immunodeficiency virus (HIV) infection. A 61-year-old man with well-controlled HIV infection presented with nephritic syndrome and palpable purpura. Laboratory evaluation revealed active urinary sediment and positive myeloperoxidase (MPO)-ANCA. Alternative causes of vasculitis were reasonably excluded based on clinical, serological, and histopathological evaluation. Skin biopsy demonstrated leukocytoclastic vasculitis, while renal biopsy showed pauci-immune necrotizing glomerulonephritis. During hospitalization, the patient developed hematochezia, for which a colonoscopy was performed, and a mass was identified in the ascending colon. Histopathological analysis confirmed stage IIIA colorectal adenocarcinoma. Treatment included glucocorticoids, plasmapheresis, and azathioprine, followed by right hemicolectomy and adjuvant CAPEOX chemotherapy. Partial improvement in the cutaneous lesions and glomerular filtration rate was observed following vasculitis-directed therapy, the patient achieved complete resolution of both the renal and cutaneous manifestations only after right hemicolectomy and adjuvant CAPEOX chemotherapy.

This case is consistent with a possible paraneoplastic link between MPO-AAV and colorectal adenocarcinoma. In patients with HIV infection, distinguishing true vasculitis from incidental autoantibody positivity is challenging. The temporal relationship and improvement with cancer-directed therapy raise the possibility that malignancy may act as a trigger for clinically overt vasculitis. Recognition of this relationship may help guide appropriate management and avoid unnecessary or prolonged immunosuppression.

## Introduction

Antineutrophil cytoplasmic antibody (ANCA)-associated vasculitis (AAV) is typically idiopathic, and its association with malignancy is uncommon. Among malignancy-associated cases of AAV, the most common cancers reported are hematologic malignancies, particularly non-Hodgkin lymphomas and myelodysplastic syndromes [1,2]. Associations with solid tumors are less frequent but have been described in bladder cancer and non-melanoma skin cancers, especially squamous cell carcinoma [3,4].

AAV may represent a paraneoplastic phenomenon resulting from tumor-induced immune dysregulation, which is characterized by abnormal lymphocyte activation and autoantibody production. Clinically, vasculitis can precede, coincide with, or follow the diagnosis of malignancy. The onset of vasculitis within 12 months of cancer diagnosis, together with resolution following cancer-directed therapy, is considered indicative of a paraneoplastic origin [5,6].

Cancer is a common complication in people living with human immunodeficiency virus (HIV), even when they receive effective antiretroviral therapy. When both conditions co-occur, they can worsen immune dysfunction, abnormal lymphocyte activity, and autoantibody production. Because of this, diagnosing AAV is especially challenging in patients with HIV infection [7]. Autoantibodies are detected in 20% to 83% of HIV-positive individuals, frequently without clinical manifestations [8,9]. Although ANCA positivity has been reported in up to 83% of cases, clinically overt vasculitis is uncommon, with an estimated incidence of less than 1% [10-15].

Distinguishing true AAV from incidental autoantibody positivity in HIV-infected patients is challenging and has significant implications for clinical management. With our case of myeloperoxidase (MPO)-AAV in a patient with well-controlled HIV infection and colorectal cancer, we want to show the diagnostic challenges and the potential paraneoplastic origin of this association.

## Case presentation

A 61-year-old male patient with a history of HIV infection, stage C3, diagnosed 16 years prior, currently with an undetectable viral load (< 20 - 50 copy/ml) and a CD4+ T-lymphocyte count of 298 cells/µL (500 - 1,500 cells/ µL), was evaluated. His past medical history was also significant for disseminated histoplasmosis and treated hepatitis B. He was receiving antiretroviral therapy with dolutegravir/lamivudine 50/300 mg daily.

He was admitted with a clinical history of approximately one year of evolution, characterized by macroscopic hematuria, lower limb edema, and intermittent palpable purpura on the trunk and extremities. On review of systems, he reported a 14 kg weight loss over two months and migratory arthralgia. On admission, blood pressure was 160/90 mmHg, and grade 2 pitting edema was noted in the lower extremities.

Laboratory evaluation revealed mild normocytic normochromic anemia on complete blood count, with iron studies consistent with iron deficiency anemia. Serum creatinine was elevated compared with baseline prior to presentation (baseline creatinine 0.9 mg/dL), with a glomerular filtration rate of 39 mL/min/1.73 m², calculated using the CKD-EPI equation, consistent with acute kidney injury. Urinalysis showed an inflammatory sediment, with hematuria and proteinuria (Table 1).

**Table 1:** Laboratory findings MCV: Mean Corpuscular Volume; MCH: Mean Corpuscular Hemoglobin; RDW: Red Cell Distribution Width; ANCA: Antineutrophil Cytoplasmic Antibodies; MPO: Mieloperoxidasa; PR3: Proteinase 3; IIF: Indirect Immunofluorescence; ANAs: Antinuclear Antibodies; Anti-dsDNA: Anti-Double-Stranded DNA (Deoxyribonucleic Acid); ENA: Extractable Nuclear Antibodies.

Parameter	Result	Reference Range
Hematology		
Hemoglobin	11.4 g/dL	12.1–16.6 g/dL
Hematocrit	36.9%	35–49%
MCV	84.4 fL	80–100 fL
MCH	30.9 pg	33–36 pg
RDW	14.2%	11.5–14.5%
Renal function		
Serum creatinine	1.91 mg/dL	0.67–1.17 mg/dL
Urinalysis		
Proteinuria (24 h)	2557.2 mg/24 h	<140 mg/24 h
Hematuria	30–50 RBC/HPF	0–5 RBC/HPF
Protein (dipstick)	500 mg/dL	
Immunology		
MPO-ANCA	83.19 U/mL	<5 mg/dL
PR3-ANCA	2.78 U/mL	<5 mg/dL
ANCA (IIF)	Positive (1:40)	Negative (<1:20)
ANA	Positive	
ANA pattern	Cytoplasmic (AMA) and reticular cytoplasmic (1:320); multiple nuclear dots (1:640)	
Anti-dsDNA	12.4 IU/mL	<20
ENA panel (SSA, SSB, RNP, Sm)	Negative	
Complement C3	84.35 mg/dL	81.1–157
Complement C4	10.6 mg/dL	12.9–39.2
Cryoglobulins	Negative	
Iron studies		
Ferritin	40 ng/mL	30–400 ng/mL
Transferrin saturation	10.04%	>20%
Serum iron	27.6 µg/dL	59–158 µg/dL

Based on the aforementioned clinical and laboratory findings, a diagnosis of nephritic syndrome was established, likely secondary to rapidly progressive glomerulonephritis, along with iron deficiency anemia. Oral iron supplementation was initiated after excluding an acute bacterial infectious process. Given the nephritic presentation, an etiological workup was undertaken to explore causes related to autoantibody-mediated disease (anti-glomerular basement membrane antibody syndrome), pauci-immune mechanisms (ANCA-associated vasculitis), and immune complex-mediated diseases (systemic lupus erythematosus, IgA nephropathy, post-streptococcal glomerulonephritis).

Diagnostic testing included antinuclear antibodies (ANAs), complement levels (C3 and C4), anti-dsDNA, extractable nuclear antigen antibodies (ENAs), ANCA, screening for chronic viral infections other than HIV (hepatitis B, hepatitis C, syphilis), serum protein electrophoresis, rheumatoid factor, and serum cryoglobulins (Table 1), following established diagnostic algorithms for nephritic syndrome and rapidly progressive glomerulonephritis.

**Figure 1:**
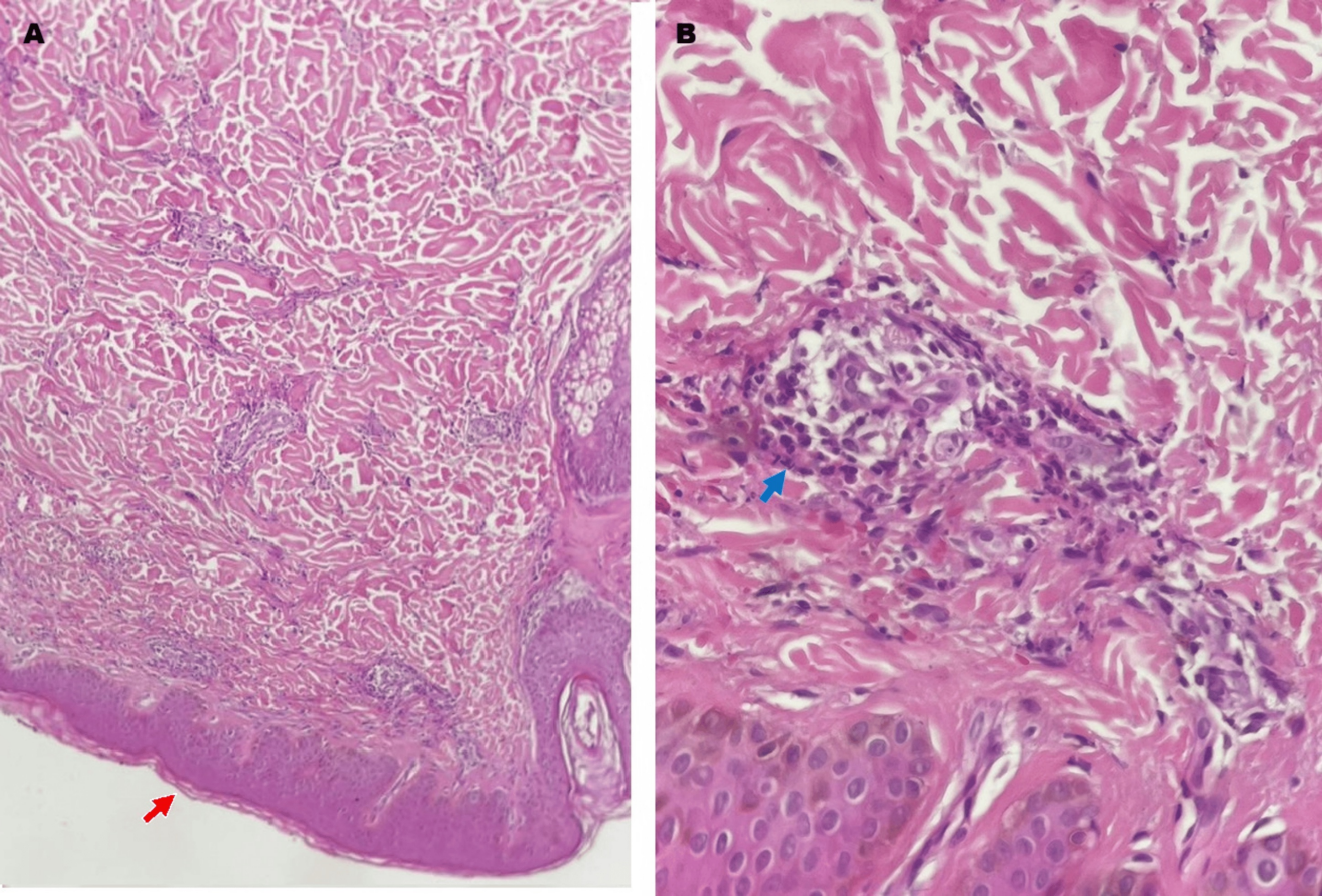
Skin biopsy A. Hematoxylin–eosin staining: orthokeratotic epidermis without significant alterations (red arrow). B. Hematoxylin–eosin staining: dermis showing edema associated with a lymphohistiocytic inflammatory infiltrate with neutrophils and scarce eosinophils, with superficial perivascular and interstitial distribution, leukocytoclasia, reactive endothelial changes, and fibrinoid necrosis of the vessel wall (blue arrow).

**Figure 2:**
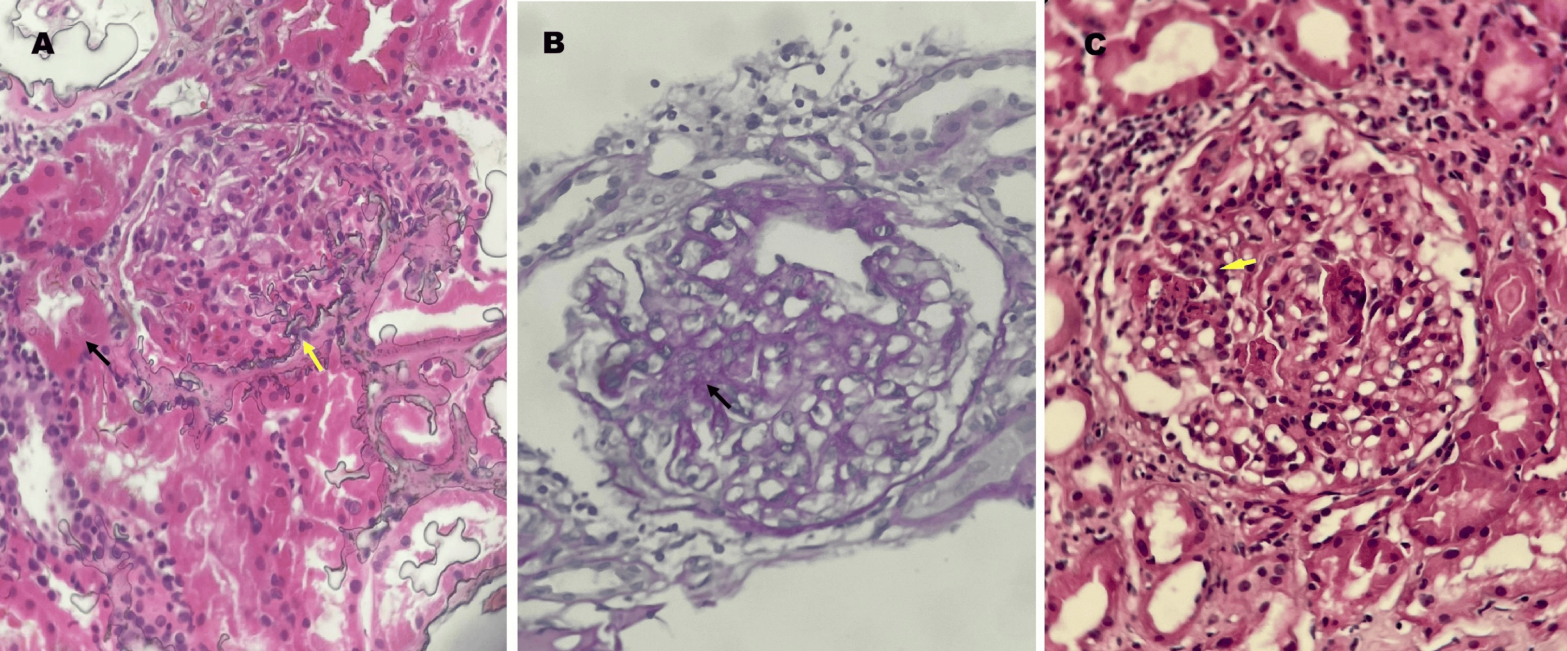
Renal biopsy A. Hematoxylin–eosin staining: glomerulus showing hypercellularity and mesangial proliferation due to increased mesangial cells within the capillary tuft (yellow arrow), along with tubulointerstitial inflammatory infiltrate consistent with tubulointerstitial nephritis (black arrow). B. Periodic acid–Schiff (PAS) staining: mild thickening of capillary walls and mesangial hypercellularity (black arrow). C. Fibrinoid necrosis (yellow arrow).

During clinical follow-up, the patient developed purpuric lesions, prompting a skin biopsy, which demonstrated leukocytoclastic small-vessel vasculitis (Figure 1). Due to nephritic involvement, a renal biopsy was performed, revealing mesangioproliferative glomerulonephritis, absence of immune complex deposition on immunofluorescence, and the presence of fibrinoid necrosis findings consistent with ANCA-associated vasculitic involvement (Figure 2). The combined findings from skin and renal biopsies, along with positive perinuclear anti-neutrophil cytoplasmic antibodies (p-ANCA) associated with MPO, supported the diagnosis of pauci-immune AAV.

During hospitalization, the patient developed hematochezia, prompting colonoscopy, which revealed a neoplastic-appearing lesion in the proximal ascending colon suggestive of malignancy. Histopathological examination confirmed a moderately differentiated ulcerated intestinal-type adenocarcinoma, NOS (Not Otherwise Specified), with pathological staging pT2pN1bM0, stage IIIA. Molecular analysis showed HER2-negative status, proficient mismatch repair, and wild-type RAS.

For renal involvement, initiation of the CYCLOPS protocol was considered [16]; however, given the suspected secondary nature of the vasculitis and the planned oncologic management including surgical intervention (radical right hemicolectomy) and adjuvant chemotherapy with the CAPEOX protocol (six cycles) [17], it was decided to manage the vasculitic process with five sessions of plasmapheresis, intravenous methylprednisolone pulses (500 mg daily for three days), followed by oral prednisolone 60 mg daily with subsequent tapering and discontinuation. Azathioprine 50 mg daily was initiated as a steroid-sparing agent. This approach resulted in partial improvement of renal function and cutaneous lesions. Subsequently, after right hemicolectomy and completion of CAPEOX chemotherapy, the cutaneous lesions resolved completely and the acute kidney injury fully recovered.

According to the classification criteria for possible cancer-associated vasculitis, this case suggests a causal relationship with the underlying malignancy. The patient had a confirmed diagnosis of cancer, clinical manifestations of vasculitis, and a total score of 9 points according to the 2022 ACR/EULAR classification criteria for AAV of the microscopic polyangiitis type (p-ANCA and MPO positivity: +6 points; pauci-immune glomerulonephritis on biopsy: +3 points) [18]. The significant improvement in renal and cutaneous involvement following antitumor treatment supports the diagnosis of AAV as a paraneoplastic manifestation, despite the coexistence of HIV infection.

## Discussion

The term “tumor-associated vasculitis” was formally introduced at the 2012 Chapel Hill Consensus Conference, where it was defined as vasculitic involvement of one or multiple organ systems induced directly or indirectly by an underlying neoplasm [19]. The 2022 recommendations from the European Alliance of Associations for Rheumatology (EULAR) established classification criteria for possible cases of cancer-associated vasculitis. These criteria include: 1) a confirmed diagnosis of malignancy; 2) clinical manifestations of vasculitis fulfilling established diagnostic criteria; 3) significant improvement of vasculitic symptoms following antitumor therapy, regardless of the temporal sequence of diagnoses; and 4) exclusion of alternative causes of vasculitis [18]. Although the causal relationship remains incompletely understood, the occurrence of vasculitis in close temporal proximity to cancer diagnosis and its improvement following oncologic treatment are considered suggestive of a paraneoplastic origin.

A key issue in this case is whether the vasculitis should be attributed to HIV-associated immune dysregulation or to a paraneoplastic phenomenon secondary to colon cancer. Several elements support a paraneoplastic contribution. First, the vasculitic manifestations developed in temporal proximity to the diagnosis of colorectal adenocarcinoma and were accompanied by significant constitutional symptoms, including marked unintentional weight loss. Second, after plasmapheresis, the patient continued treatment with prednisolone and azathioprine, which were administered concomitantly with cancer-directed therapy. Complete improvement of cutaneous and renal manifestations was ultimately observed following surgical resection and systemic oncologic therapy, suggesting a relationship between tumor burden and immune activation. Third, the detection of MPO-ANCA, which is more frequently associated with clinically relevant small-vessel vasculitis, supports a pathogenic rather than incidental serological finding [20].

It is plausible that, in this patient, HIV-related immune dysregulation created a background of chronic polyclonal B-cell activation and autoantibody production, while tumor-associated immune activation acted as a “second hit,” promoting the transition from subclinical autoimmunity to clinically overt vasculitis. This case highlights the diagnostic challenge posed by ANCA positivity in patients with HIV infection. Given the high prevalence of non-pathogenic autoantibodies in this population, clinicians must carefully distinguish incidental serological findings from true systemic vasculitis [8]. The presence of compatible clinical features together with histopathological confirmation remains essential.

Table 2 provides a comparative overview of the clinical, serological, and pathophysiological characteristics of AAV as a paraneoplastic syndrome versus AAV in the context of HIV infection, highlighting their differences.

**Table 2:** Key characteristics of ANCA-associated vasculitis as a paraneoplastic syndrome vs ANCA-associated vasculitis in HIV [5,9,11] AAV: Antineutrophil cytoplasmic antibody-associated vasculitis; MPO: myeloperoxidase

Feature	Paraneoplastic AAV	HIV-associated AAV
Frequency	Rare; described in association with malignancy	Very rare; ANCA positivity common, but clinical vasculitis uncommon
Underlying mechanism	Tumor-driven immune dysregulation with autoantibody production	HIV-related immune dysregulation with polyclonal B-cell activation
Temporal relationship	May precede, coincide with, or follow cancer; close temporal association supports causality	No consistent temporal pattern; may occur at any stage of HIV
ANCA profile	Typically clinically relevant (often MPO-ANCA), no precise prevalence has been described in the literature	Frequently positive 20%, but often nonspecific
Clinical presentation	Systemic involvement; may be atypical or subacute	Often asymptomatic; when present, usually limited (cutaneous or renal)
Renal involvement	Common; pauci-immune glomerulonephritis	Uncommon; variable presentation
Response to immunosuppression	Often incomplete without treatment of underlying malignancy	Variable; limited by risk of infection
Impact of etiological treatment	Improvement or resolution after cancer treatment	Not applicable

## Conclusions

AAV as a paraneoplastic manifestation is an uncommon entity, particularly in the context of solid tumors. In patients with HIV infection, the presence of ANCA represents a diagnostic challenge due to the high prevalence of autoantibodies without clinical correlation.

This case highlights the importance of considering a paraneoplastic etiology in patients with atypical or refractory presentations of AAV, even in the presence of HIV infection, as malignant neoplasms may coexist. In our case, the clinical improvement of renal and cutaneous involvement following surgical and oncologic treatment of colon cancer strongly supports the paraneoplastic origin of the vasculitis. Early recognition of this association allows for a therapeutic approach directed at the underlying cause, avoiding unnecessary immunosuppression and improving patient outcomes.
